# Rethinking Short-Chain Fatty Acids: A Closer Look at Propionate in Inflammation, Metabolism, and Mucosal Homeostasis

**DOI:** 10.3390/cells14151130

**Published:** 2025-07-22

**Authors:** Sonia Facchin, Matteo Calgaro, Edoardo V. Savarino

**Affiliations:** 1 Department of Surgery, Oncology and Gastroenterology (DISCOG), University Hospital of Padua, 35128 Padua, Italy; sonia.facchin@unipd.it; 2 Department of Biotechnology, University of Verona, 37134 Verona, Italy; matteo.calgaro_01@univr.it

**Keywords:** short-chain fatty acids, metabolites, microbiota, IBD, IBS

## Abstract

Propionate is a short-chain fatty acid (SCFA) produced by gut microbiota through the fermentation of dietary fibers. Among the SCFAs, butyrate stands out and has been extensively studied for its beneficial effects; however, propionate has received less attention despite its relevant roles in immune modulation, metabolism, and mucosal homeostasis. This narrative review focuses on propionate’s effects on metabolism, inflammation, microbiota, and gastrointestinal diseases. Propionate acts as a signalling molecule through FFAR2/FFAR3 receptors and modulates immunity, energy metabolism, and gut–brain communication. It has beneficial effects in metabolic disorders, inflammatory bowel disease (IBD), and alcohol-related liver disease (ALD). However, excessive accumulation is linked to neurotoxicity, autism spectrum disorder (ASD), and mitochondrial dysfunction. Its effects are dose-dependent and tissue-specific, with both protective and harmful potentials depending on the context. Propionate use requires a personalized approach, considering the pathological context, host microbiota composition, and appropriate dosage to avoid adverse effects.

## 1. Introduction

Short-chain fatty acids (SCFAs), produced through the fermentation of dietary fibers by the intestinal microbiota, are generating increasing interest in scientific research [1,2]. Among these lipid molecules, propionate emerges due to its multiple physiological effects and therapeutic potential in various pathological conditions. With the advent of the “microbiota revolution” and advances in next-generation sequencing and bioinformatics tools, a better understanding of the crucial role played by SCFAs, such as propionate, in regulating the microbiota–host axis has emerged [3,4].

Among all SCFAs, butyrate has been the most studied so far due to its beneficial effects on intestinal mucosa and homeostasis [5,6], while propionate has often been underestimated. However, recent reviews have emphasized the synergistic role of acetate and propionate in the production of butyrate and the modulation of key processes such as inflammation, metabolism, and mucosal barrier homeostasis [1,2].

In particular, low levels of SCFAs, including propionate, have been associated with various diseases such as type 1 and 2 diabetes, liver cirrhosis, chronic inflammatory diseases, modulation of adipose tissue metabolism to influence whole-body metabolic processes, and other conditions [7,8,9,10]. However, excessive systemic accumulation of propionate has been linked to neurotoxicity and mitochondrial dysfunction, highlighting the importance of dose and indication. Nevertheless, the current medical literature suggests that restoring physiological levels of propionate could represent a promising therapeutic approach for improving various symptoms and disorders.

From an integrative therapy perspective, formulations of propionate used for supplementation have been rarely investigated in clinical trials (particularly in the context of obesity, diabetes, and cardiovascular disease) [1]. Therefore, evaluating these formulations is crucial for future therapeutic applications.

This narrative review aims to provide a comprehensive overview of the current scientific evidence regarding the distinctive biochemical and physiological properties of propionate, with particular emphasis on its therapeutic potential in the gastroenterological field. The implications of propionate in intestinal and systemic inflammatory diseases will be explored, and it will be shown, based on the data at our disposal, how a careful evaluation of the dosage of propionate is necessary in relation to the symptoms and pathology to be treated.

## 2. Materials and Methods

This narrative review was conducted after a thorough literature search using various biomedical databases, including PubMed, Scopus, Web of Science, and Google Scholar. Keywords used for the search included “propionate”, “short-chain fatty acids”, “gut microbiota”, “inflammation”, “immunity”, “inflammatory bowel disease”, “irritable bowel syndrome”, “alcoholic liver injury”, “metabolic diseases”, “gut-brain axis”, “skin”, and combinations thereof.

Primary studies, systematic reviews, and meta-analyses focusing on the role of propionate in gastrointestinal and systemic physiology and pathology, published in the last 10 years in peer-reviewed journals, were included. In addition, seminal reference articles from earlier years were considered if relevant to the understanding of the mechanisms of action and biochemical properties of propionate.

In selecting studies, the following inclusion criteria were applied: (1) in vivo studies from animal or human models; (2) in vitro studies in relevant cell lines; (3) studies investigating the effects of propionate on the microbiota, inflammation, immunity, or metabolism; and (4) studies evaluating the therapeutic potential of propionate in inflammatory bowel disease (IBD), irritable bowel syndrome (IBS), alcohol-induced liver damage (ALD), or other diseases.

Studies that were not available in English, that did not meet the inclusion criteria, those with inadequately described methodologies, or those with unclear or contradictory results were excluded.

Relevant data were extracted from the selected studies, including information on experimental designs, study populations, interventions, outcome measures, and main conclusions. These data were summarized and organized into thematic sections to facilitate critical analysis and integration of evidence from different sources.

## 3. Results

### 3.1. Biosynthesis and Metabolism of Propionate

Acetate, butyrate, and propionate are lipid molecules, consisting of a chain of one to six carbon atoms, produced mainly through bacterial fermentation of dietary fibers in the colon. They have a simple structure; however, they act as sophisticated messengers between the intestinal microbiota and the organism, modulating inflammation, metabolism, and the balance of the intestinal barrier [11].

Propionate, together with butyrate and acetate, is produced in the colon and distal small bowel through the fermentation of dietary fibers, resistant starch, and poorly digestible polysaccharides by the microbiota. The intestinal origin of propionate has been confirmed by many studies showing that propionate levels in the portal vein are about 50 times higher than those in circulating plasma in a study on mice [12]. Further evidence comes from germ-free mice, which show extremely low (70-fold lower) levels of propionate in the portal vein compared to control mice, strongly indicating that the majority of propionate is derived from the microbiome [12]. For a 70 kg human, the gut microbiome produces approximately 2 g of propionate per day [13,14], with concentrations reaching up to 10–30 mM in the proximal colon [15].

The Human Metabolome Database (http://www.hmdb.ca/ (accessed on 1 January 2025)) reports blood, cerebrospinal fluid, and gut propionate concentrations (see Table 1).

**Figure 1:**
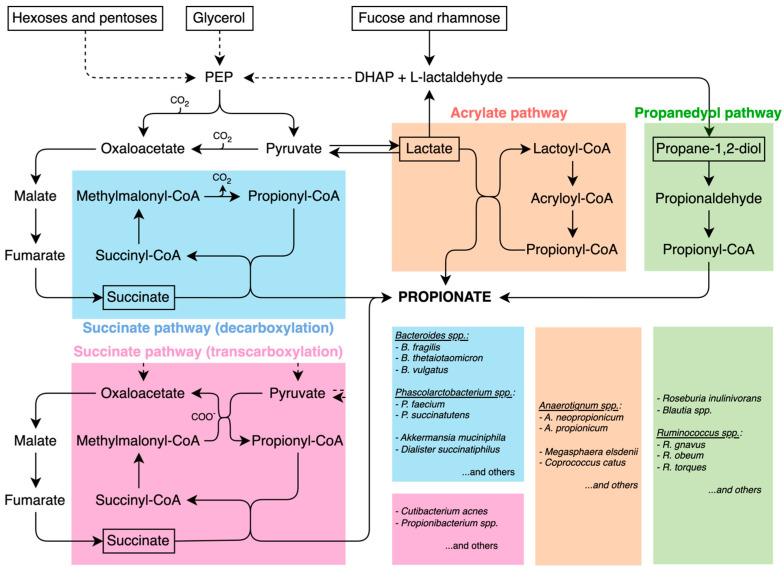
Propionate pathways: succinate (in two variants) (blue and pink), acrylate (orange), and propanediol (green) pathways. The boxes on the right report the main bacterial taxa utilizing the pathways indicated by the corresponding colors.

Four main pathways have been proposed in the gut for propionate formation, namely, succinate (in two variants), acrylate, and propanediol pathways (see Figure 1, blue and pink, orange, and green panels, respectively). The first is described as the succinate or randomizing pathway [15,17], in which propionate is formed via the decarboxylation of succinate. Fermentable fibers are first converted into pyruvate, which is carboxylated to oxaloacetate, then reduced to malate, dehydrated to fumarate, and further reduced to succinate. Finally, propionate is generated through succinate metabolism, involving intermediate steps such as the formation of propionyl-CoA and its subsequent decarboxylation via the methylmalonyl-CoA pathway. This route of production of propionate is typical of *Bacteroides* spp. such as *B. fragilis*, *B. vulgatus*, *B. thetaiotaomicron*, and others and is the most widely used by intestinal bacteria that produce propionate.

A second variant of the succinate pathway is the Wood–Werkman cycle, which relies on transcarboxylation reactions catalyzed by methylmalonyl-CoA transcarboxylase enzymes. Unlike the classical succinate pathway, which releases CO_2_, this cycle conserves carbon atoms by transferring carboxyl groups via methylmalonyl-CoA transcarboxylase enzymes, making it the most energy-efficient route of propionate fermentation [18]. Microorganisms capable of this type of fermentation include *Cutibacterium acnes*, which is a predominant bacterial species on the surface of the skin [19].

A third production mechanism has been observed, namely, the acrylate route. If in the previous routes, pyruvate has formed oxaloacetate by pyruvate carboxylase or methylmalonyl-CoA transcarboxylation, another enzyme, lactate dehydrogenase, reduces pyruvate to lactate, which is converted to Lactyl-CoA and dehydrated to Acryl-CoA, subsequently reduced to propionyl-CoA to reach propionate, after some other metabolic steps. The acrylate pathway is typical of *Anaerotignum neopropionicum* [20] (former *Clostridium neopropionicum*) and *Megasphaera elsdanii* [21]. The former is found primarily in the gut of ruminants such as sheep and cattle, but also in the human intestine, and preferentially utilizes lactate over glucose for propionate production. The latter resides mainly in the rumen of ruminant animals where it plays a key role in fermentation. Some *Megasphaera* species are also part of the human vaginal microbiota [22,23]. Although the acrylate pathway itself is a microbial fermentation route, the propionate it generates can be absorbed by the host and converted into propionyl-CoA, which then acts as an anaplerotic substrate, replenishing tricarboxylic acid (TCA or Krebs cycle) cycle intermediates when cataplerosis depletes them [24,25]. The metabolic flux of propionyl-CoA entering the Krebs cycle is significantly slower than that of acetyl-CoA [12]. However, anaplerosis is essential in organs with high cataplerotic activity, such as the liver, kidney, intestine, pancreas, and brain [26,27,28,29,30]. Furthermore, propionate supplementation has been shown to provide benefits beyond its role in anaplerosis, such as reducing lipogenesis, lowering serum cholesterol levels, attenuating depressive-like behaviours, and reducing the risk of carcinogenesis [17].

The fourth propionate production pathway is the propanediol route, which metabolizes deoxy sugars such as rhamnose and fucose. This pathway has been identified in the *Proteobacterium Salmonella enterica Typhimurium*, as well as in members of the *Lachnospiraceae* family, including *Blautia* spp., *Ruminococcus obeum*, and *Roseburia inulinivorans* [31,32].

A compendium of propionate producers is given in Table 2.

### 3.2. Mechanism of Action of Propionate

#### 3.2.1. Interaction with Receptors (FFAR2 and FFAR3) and Hormonal Effects

Once absorbed, propionate is mainly used by the liver, acting as a signaling molecule, showing particular agonism on both FFAR2 (GRP43) and FFAR3 (GRP41) receptors, expressed on epithelial cells, adipose tissue, and immune cells such as neutrophils, dendritic cells, macrophages, and lymphocytes. It has been observed that propionate, through FFAR2, directly influences brain endothelial cells. In in vitro studies, on human brain endothelial cells (hCMEC/D3), propionate increased the expression of tight junction proteins, such as occludin and zonulin (ZO-1), contributing to the maintenance of the blood–brain barrier [54]. In the intestinal endothelial tissue, these receptors are co-localized with enteroendocrine cells that express peptide yy (PYY)—a hormone produced by L-cells in the intestine, particularly in the ileum and colon, and is released after meals. Its main function is to reduce appetite and slow down gastrointestinal motility, contributing to the feeling of satiety. This co-localization has led some authors to examine the effect of SCFA on intestinal peptides and other hormones [55], demonstrating that, in rodents, the oral administration of sodium propionate (400 mg/kg) produced a significant increase in plasma levels of glucose-dependent insulinotropic polypeptide (GIP), insulin, and amylin within 10 min of product intake. In parallel, it did not significantly increase the levels of glucagon-like peptide 1 (GLP-1 is an intestinal hormone that stimulates insulin secretion, inhibits glucagon production, slows gastric emptying, and increases the feeling of satiety, helping to regulate blood glucose levels) or PYY (unlike butyrate). The authors argue that the hormonal profile induced by propionate suggests the stimulation of pancreatic secretion (insulin and amylin) but with a limited impact on the secretion of intestinal anorexigenic peptides. This result would be consistent with its capacity to inhibit food intake observed in mice and with the improvement of glucose tolerance, showing for propionate a distinct hormonal and metabolic profile that positions it as an intermediate modulator between acetate and butyrate in terms of anorexigenic effect. In a recent study conducted in humans [56], oral supplementation with inulin–propionate (IPE) was proposed compared to inulin alone, at a dose of 20 g/day for 42 days. IPE improved insulin resistance (to a comparable extent to inulin alone) and reduced the systemic inflammatory response, linked to insulin resistance, reducing circulating levels of IL-8, also selectively modifying the composition of the intestinal microbiota, suggesting an inhibitory effect on bifidogenesis, reducing fecal bacterial diversity, compared to cellulose, but without observable negative metabolic effects. These authors also concluded that propionate may represent a useful nutritional strategy to improve glucose homeostasis, inflammation, and immunity in subjects at metabolic risk. Recently, it has been observed that oral supplementation of propionate could modulate the serum metabolome, impacting circulating bile acids [57].

The role of propionate is multifactorial; it acts as a metabolite with significant physiological and metabolic implications at the level of the liver, where it is primarily utilized, but also at the level of the nervous system and the intestine. Overall, these data support the hypothesis that propionate possesses distinct metabolic and immunomodulatory properties, also in relation to acetate and butyrate

#### 3.2.2. Epigenetic Effects and Microbiota Modulation

The effect of propionate during caloric restriction has recently been investigated [58]. It was observed that there is a reduction in the concentration of propionate in the colonic lumen, with a consequent increase in pH favourable to the mucolytic activity of *Akkermansia muciniphila*. The administration of acidified propionate in mice subjected to caloric restriction prevented the expansion of *Akkermansia* and the thinning of the colonic mucosa, indicating that the presence of propionate can modulate the mucolytic activity of *Akkermansia*.

Propionate exhibits clear immunological effects: studies conducted on myeloid cells have demonstrated that propionate, along with butyrate, acts directly on dendritic cells, enhancing their immunoregulatory properties. It inhibits the maturation of dendritic cells induced by lipopolysaccharides (LPS), derived from human monocytes in vitro, reducing interleukin-12(IL-12) production and limiting the activation of cytotoxic T lymphocytes (CD8+ T cells) [59].

It has been observed that a category of T lymphocytes-gamma-delta (γδ) T cells, innate cells that represent a subset of interleukin-17 (IL-17)-producing T lymphocytes, are essential for the protection of the epithelial barrier following bacterial infections such as *Listeria* and *Clostridioides difficile* [60,61,62]. However, they may induce inflammation in an unclear manner in chronic intestinal inflammation [63,64], while simultaneously promoting progression to colorectal cancer [65,66]. In this context, propionate appears to reduce the production of IL-17 and IL-22 by intestinal γδ T cells through the inhibition of histone deacetylase (HDAC), likely involving HDAC1-3 [67]. Since HDAC is an intracellular target, propionate likely enters γδ T cells via passive diffusion across the plasma membrane, as is the case for macrophages. This is supported by evidence showing that the MCT1 transporter, present on γδ T lymphocytes, does not interact with propionate [68]. On the other hand, it has also been demonstrated that propionate can promote the expansion of innate lymphoid cells (ILC3) producing IL-22 in the colon, through the GRP43 signal [69].

Recently, Wang et al. explored the role of short-chain fatty acids (SCFAs), particularly butyrate and propionate, as microbial danger signals that activate the NLRP3 inflammasome in human macrophages under inflammatory conditions. These SCFAs are generally considered beneficial for intestinal health. However, in inflammatory conditions, such as in inflammatory bowel disease (IBD), they can have opposing effects. Specifically, the researchers concluded that propionate appears to act as a microbial danger signal—represented by viability-associated pathogen-associated molecular patterns (vita-PAMPs)—under inflammatory conditions. Its ability to activate NLRP3 and reduce the secretion of anti-inflammatory cytokines like IL-10 may contribute to the pathogenesis of IBD, especially when the intestinal barrier is compromised [70,71].

At the systemic level, studies conducted in vancomycin-treated mice showed that propionate concentrations in the cecum and feces were not reduced compared to control mice, unlike butyrate, which was instead depleted. The same applies to serum propionate levels [72].

Propionate also exerts important effects on skin keratinocytes through its ability to inhibit specific histone deacetylases (HDAC8/9) [73]. This epigenetic mechanism underlies the modulation and innate immune tolerance of the skin. Propionate acts on the epithelium by modifying the keratinocyte epigenome via HDAC8/9 inhibition, thereby unlocking the expression of MAP2K3. This gene is upregulated in inflammatory skin conditions such as psoriasis, atopic dermatitis, acne, and in response to UV radiation, contributing to a pro-inflammatory environment. As a result, the epithelium becomes more susceptible to reacting to stimuli that are normally tolerated, contributing to skin inflammation.

A well-known producer of propionate is *Cutibacterium acnes* (formerly Propionibacterium acnes), a dominant commensal bacterium in human pilosebaceous follicles. *C. acnes* ferments glycerol derived from skin sebum and produces propionate, which accumulates in hair follicles, increases histone acetylation by inhibiting HDAC8/9, and enhances the expression of inflammatory genes, including MAP2K3. This leads to epithelial sensitization to inflammatory stimuli recognized by Toll-like receptors (TLRs).

The pro-inflammatory role of propionate produced by *C. acnes* also represents an ecological strategy used by the bacterium to dominate the follicular microbiota at the expense of other species. In seborrheic dermatitis, for example, an increase in *Malassezia* and a decrease in *C. acnes* is observed. A recent study showed that the use of specific probiotics producing antimicrobial peptides, including propionate, strongly inhibited both *Malassezia* furfur and *C. acnes*, although with different effects depending on the combination and dose [74].

The overall scientific evidence suggests that propionate exerts a broad range of biological actions, spanning from the maintenance of intestinal mucosal integrity to the modulation of both innate and adaptive immunity, at local and systemic levels. It emerges as a multifunctional epigenetic and immune modulator, whose activity varies depending on the target tissue, microbial context, and physico-pathological state. Its ability to influence intestinal, immune, and skin homeostasis positions it as a key metabolite in the dialogue between diet, microbiota, and the immune system, with potential therapeutic implications in metabolic, inflammatory, and dermatological disorders.

### 3.3. Physiological Effects of Propionate

#### 3.3.1. Inflammation, Immunity, and Neurological Disease

It is well known that in neurodegenerative diseases (ND), short-chain fatty acids (SCFAs) are considered important factors influencing the homeostasis of the central nervous system [1]. In a study conducted in the context of Alzheimer’s disease (AD), using a mouse model, cultured cells, and elderly patients, it was observed that commensal bacteria such *Akkermansia muciniphila* may exert a protective effect against the development of neurodegeneration [75]. The authors propose that cognitive decline in the AD mouse model can be alleviated solely by restoring mitochondrial dynamics and autophagy homeostasis, without directly targeting amyloid peptide or tau protein. These findings appear to contrast with those of Schwabkey et al., who reported that propionate administration reduced elevated levels of *Akkermansia muciniphila*, preserved the intestinal mucus layer from excessive degradation, and prevented neutropenic fever in patients undergoing hematopoietic stem cell transplantation [58]. This apparent contradiction may be explained by a lack of regulatory feedback involving propionate and the vagus nerve. Recent evidence has shown that activation of the vagus nerve can influence the abundance and activity of the gut microbiota in ways that modulate SCFA production [76,77]. For example, vagus nerve stimulation may increase acetylcholine release, which in turn could affect microbiota composition, including bacteria such as *Akkermansia* [78].

The absence of a negative feedback signal to control *Akkermansia muciniphila*, despite propionate production, could be due to dysfunction in the vagus nerve regulatory system. This system may fail to detect excess SCFAs properly. As a result, in some individuals, *Akkermansia* continues to grow even when propionate levels become potentially harmful. However, in the rare disease known as propionic academia (PA)—a recessive metabolic disorder caused by mutations in the Propionyl-CoA Carboxylase Subunit Alpha (PCCA or PCCB genes)—impaired metabolism of propionyl-CoA leads to a variety of metabolic disorders. Propionate accumulation and blockage of its metabolism have synergistic detrimental effects on various organs, with neurological dysfunction being one of the most common complications in PA patients [79].

Although still a matter of debate, the potential role of propionate in autism spectrum disorder (ASD) remains a frequently cited topic. It has been proposed that alterations in the gut microbiota may influence both the onset and severity of ASD, primarily through an increased abundance of propionate-producing bacteria and a concurrent reduction in butyrate-producing species [80,81]. The propionate-induced autism model is now widely recognized as a valuable tool for studying the disorder. Administration of high doses of propionate—via subcutaneous, intragastric, intraperitoneal, or intracerebroventricular routes—has been shown in rodents to trigger marked microglial activation, increased production of neurotoxic cytokines, genetic expression alterations, structural changes in the hippocampus, and behavioural abnormalities characteristic of the autism spectrum, such as repetitive actions and impaired social interaction [82]. Finally, a clinical study conducted in China compared fecal propionate levels between children with typical autism spectrum disorder (TD) and those with ASD accompanied by gastrointestinal symptoms—specifically constipation (c-ASD). Children in the c-ASD group exhibited higher fecal propionate levels than those in the TD group, and these levels were positively correlated with symptom severity [83]. An interesting review by Killingsworth et al. suggested that high levels of propionate, along with an increase in propionate-producing bacteria, may contribute to the development of dementia, particularly Alzheimer’s disease. The main cause of elevated endogenous propionate levels is thought to be an overrepresentation of the phylum *Bacteroidetes*, major SCFA producers in the human gut, which are known to increase with age [14]. However, the latter remains a pure hypothesis.

#### 3.3.2. Energy Metabolism, Body Weight, Gastrointestinal Motility, and the Gut–Brain Axis

The multiple physiological effects of propionate become particularly relevant in the context of the gastrointestinal environment. In particular, this metabolite has been shown to contribute to the improvement of energy metabolism, promoting the reduction in fat mass and body weight [56,84]. This effect is supported by several factors, as suggested by Chen et al. [15]: (i) the activation of intestinal gluconeogenesis mediated by the gut–brain axis, involving the short-chain fatty acid receptor FFAR3; (ii) increased intestinal concentrations of propionate associated with reduced stress levels in mouse models [85]; (iii) the hypolipidemic and hypocholesterolemic action of propionate, achieved through the competitive inhibition of hepatic acetate uptake and subsequent reduction in endogenous cholesterol synthesis [86,87]; and (iv) regulation of appetite and energy expenditure through stimulation of the anorexigenic hormones PYY and GLP-1, secreted by enteroendocrine cells. This effect is mediated by the activation of G-protein coupled receptors, promoting satiety, reducing caloric intake, and supporting weight loss [14,28,87].

These data confirm that propionate acts as a critical modulator of gut–brain interaction, with both immunological and neurological implications. It is essential to carefully monitor and characterize the specific types of pathologies being targeted for treatment.

### 3.4. Therapeutic Potential in Gastrointestinal Diseases

We have repeatedly highlighted that the FFAR2 receptor (GPR43) is one of the ligands for propionate, which acts as a powerful ligand, playing a key role in modulating not only intestinal inflammation but also neutrophil recruitment and cytokine production. It has been observed that propionate can influence NF-kB signalling, which is involved in the regulation of blood–brain barrier (BBB) permeability [88]. However, its therapeutic potential may depend on the appropriate therapeutic dosage of propionate (Table 3). In animal model studies, high doses (750 mg/kg of body weight) were shown to exert neurotoxic effects while low doses (2 mg/kg body weight/day) demonstrated antidepressant effects [89,90]. In human studies that demonstrated efficacy, the therapeutic dose was approximately 0.15–0.30 mg/kg body weight [56,84,91]. Nevertheless, the effects may vary depending on the experimental model and are not always exclusively GPR43-dependent. Thus, propionate appears to be a potential therapeutic agent for maintaining BBB integrity, particularly under conditions of gut dysbiosis [88], as well as in IBD [92].

Propionate-producing probiotic strains such as *Propionibacterium freudenfeichii* B-11921 or Propionibacterium jensenii B-6085, *Propionibacterium freudenreichii* B-11921, Propionibacterium thoenii B-6082, and *Propionibacterium acidipropionic* B-5723 have been studied for their biocompatibility in the development of bacterial consortia potentially useful in the correction of dysbiosis in chronic diseases such as diabetes, obesity, atherosclerosis, and inflammation [93]. However, it is important to note that the potential benefit of propionate in chronic inflammatory bowel diseases may not be directly translatable to irritable bowel syndrome.

A different evaluation of propionate’s effect has emerged in IBS, one of the most common chronic gastrointestinal disorders. IBS significantly reduces quality of life and work productivity [94,95]. It has been observed that fecal concentrations of commensal-derived-propionate were significantly higher in patients compared to healthy controls, particularly in diarrhea-predominant IBS patients (SMD = 0.32, 95% CI: 0.12–0.51) [96]. This increase in propionate may be due to a greater presence of *Lactobacillus* and *Veillonella* [97,98], known propionate producers. Moreover, a role for propionate has been proposed in the activation of subcortical brain circuits involved in pain perception and visceral stress responses [99]. IBS patients undergoing dietary therapy with a Low-FODMAP (Fermentable, Oligosaccharides, Disaccharides, Monosaccharides, and Polyols) diet reported a significant reduction in fecal propionate levels, suggesting that this diet may normalize SCFA alterations in IBS patients by reducing propionate levels [96]. Therefore, the analysis of fecal propionate concentration may be considered a useful biomarker for IBS diagnosis, especially for the diarrheal subtype. Recent studies have identified *Collinsella aerofaciens* as a biomarker in non-diarrheal IBS patients [100]. Interestingly, *C. aerofaciens* is a major lactate producer [101], and through bacterial cross-feeding, lactate can be transformed into propionate by lactate-consuming genera such as *Veillonella* spp. [102]. Of course, this observation remains speculative and should be confirmed by further data.

In ALD, supplemented propionate has demonstrated the ability to prevent liver function loss and reduce steatosis by exerting a protective effect on the intestinal barrier and regulating the gut microbiota. The authors highlighted that the increase in propionate levels in intestinal contents was observed after supplementation, whereas no increase was detected in the liver, suggesting that the intestine is the primary site of propionate action [103]. The same authors evaluated the effect of propionate supplementation on the microbiota and observed restoration of microbial diversity, normalization of the Firmicutes/Bacteroidetes ratio, and reduction in pathogenic abundance, alongside the restoration of beneficial bacteria. They propose propionate as a potential therapeutic candidate for ALD, demonstrating a direct action through the restoration of gut–liver axis homeostasis.

Overall, these findings support the potential of propionate as a multifunctional therapeutic agent, whose effects are dose-dependent, tissue-specific, and strongly influenced by the gut microbiota. However, clinical use of propionate requires caution and personalization, considering its functional ambivalence, with either beneficial or harmful effects depending on the pathological context, dosage, and host microbiota composition (Table 4).

## 4. Conclusions

Propionate has proven itself as a key metabolite in the regulation of immune responses, intestinal and brain barrier function, and in the modulation of the microbiota across different pathophysiological contexts. However, its impact is highly dependent on concentration, pathological context, and neuro-enteric regulation, underlining the need for personalized therapeutic approaches that consider microbiota composition, age, and the functional state of the vagus nerve. These findings open new perspectives for the controlled use of propionate in neurological and immunological contexts, particularly in neurodegenerative and autoimmune diseases.

## Figures and Tables

**Table 1:** Propionate concentrations in blood, cerebrospinal fluid, and the gut.

Propionate Concentrations [13]		
Blood	0.9 ± 1.2 μM	^b^ (Lentner, 1981)
Cerebrospinal Fluid	2.8 ± 3.2 μM	^b^ (Lentner, 1981)
Gut (wet feces)	6.58 − 14.4 μmol/g	^a^ (Han et al., 2015)
	11.4 ± 4.74 μmol/g	^a^ (Zheng et al., 2013)
	12.5 (4.5–27.8) μmol/g	^a^ (Høverstad et al., 1984)

^a^: References collected from the Human Metabolome Database (https://hmdb.ca/ (accessed on 1 January 2025)). ^b^: References collected from the *Geigy Scientific Tables* [16].

**Table 2:** Table of propionate-related microbes.

Bacterium	Role in Propionate Production	Pathway	Body Site/Source
***Akkermansia muciniphila* [33]**	Mucin degrader in the colon; converts succinate to propionate, requiring vitamin B_12_ as a cofactor	Succinate	Human gut (intestinal mucosa)
***Dialister succinatiphilus* [34,35]**	Consumes succinate produced by others; converts succinate to propionate through succinate decarboxylation	Succinate	Human gut (feces)
***Phascolarctobacterium faecium*, *P. succinatutens* [36]**	Uses succinate as a sole energy source to produce propionate	Succinate	Human gut (feces)
***Veillonella* spp., *V. parvula*, *V. Alcalescens* [37]**	Ferments lactate into propionate and acetate via methylmalonyl-CoA	Succinate	Oral cavity and human gut
***Bacteroides* spp., *B. fragilis*, *B. vulgatus*, *B. thetaiotaomicron* [38]**	Convert succinate to propionate via succinyl-CoA and methylmalonyl-CoA	Succinate	Human gut (feces)
***Prevotella copri* [39]**	Mainly produces succinate; propionate not direct in most strains	Succinate	Human gut (feces)
***Prevotella ruminicola*, *P. brevis* [40]**	Produces propionate when vitamin B_12_ is available in the rumen	Succinate	Rumen of cattle, sheep, and goats
***Propionigenum modestum* [41]**	It uses a sodium-translocating methylmalonyl-CoA decarboxylase complex	Succinate	Intestinal tract
***Selenomonas ruminantium* [42]**	Major ruminal propionate producer; stabilizes the rumen via succinate–propionate fermentation	Succinate	Rumen of cattle, sheep, and goats
***Cutibacterium acnes* [43,44,45]**	Efficiently generates propionate through an ATP-independent route from glycerol or glucose	Succinate (transcarboxylase)	Skin (sebaceous glands) and human gut
***Acidipropionibacterium acidipropionici (former Propionibacterium acidipropionici) Propionibacterium freudenreichii*, *P. jensenii*, *P. thoenii* [46]**	Optimized propionate production, key in cheese ripening, industrial fermentation, probiotics	Succinate (transcarboxylase)	Cheese/fermented dairy products
***Roseburia inulinivorans* [33,47]**	Substrate-induced propionate producer (only on fucose via the propanediol pathway)	Propanediol	Human gut (feces)
***Blautia* spp. [48,49]**	Uses fucose/rhamnose to generate propionate via propanediol operon	Propanediol	Human gut (feces)
***Ruminococcus gnavus*, *R. obeum* [33,50]**	Ferments deoxy-sugars into propionate via propanediol pathway	Propanediol	Human gut (feces)
***Ruminococcus torques* [51]**	Certain strains produce propionate when grown on mucin and fucosylated glycans	Propanediol	Intestinal tract
***Escherichia coli* [52]**	Can produce propionate as a secondary metabolite under specific conditions	Propanediol (partial)	Part of the propionate-producing consortia
***Salmonella enterica* (Typhimurium) [31,32]**	Can produce 1,2-propanediol from rhamnose/fucose; often releases it without full conversion	Propanediol (partial)	Human gut (faces)
***Megasphaera elsdenii* [21,22,23]**	It plays a critical role in preventing lactate accumulation by converting lactate into propionate	Acrylate	Rumen of cattle, sheep, and goats
***Anaerotignum neopropionicum and propionicum (former Clostridium neopropionicum and propionicum)* [53]**	Specialized role in converting substrates like ethanol and lactate into propionate under anaerobic conditions	Acrylate	Industrial wastewater and animal feces
***Coprococcus catus* [33]**	Ferments ethanol/lactate to propionate via the acrylate pathway	Acrylate	Human gut (feces)

**Table 3:** Propionate Dose-Dependence.

Propionate Dosage	Observed Effect	Study Design
High (750 mg/kg body weight)	Neurotoxic	Animal model [89]
Low (2 mg/kg body weight)	Antidepressant	Animal model [90]
10 gr/day * IPE;	Improvement	H-DB-RCT in the obese [84]
20 gr/day * IPE;	Improvement	H-DB-RCT crossover in the obese [56]
1 gr/die * propionic acid	Improvement	H-DB-RCT in ACVD [91]

* Adult of 70 kg, IPE = inulin–propionate ester; H = human; DB-RCT = Double Blind-Randomized Clinical Trial; ACVD = atherosclerotic cardiovascular disease.

**Table 4:** Clinical decision-making.

Clinical Context	Recommendation
**Inflammatory Bowel Disease (IBD)**	✅ May be considered, with appropriate monitoring
**Obesity and Dyslipidemia**	✅ May be considered
**Alcoholic Liver Disease (ALD)**	✅ May be considered
**Hematopoietic Stem Cell Transplantation** **(HSCT)**	✅ May be considered
**Irritable Bowel Syndrome** **(particularly IBS-D)**	🚫 Should be avoided
**Autism Spectrum Disorder (ASD)**	🚫 Should be avoided
**Propionic Acidemia (PA)**	🚫 Contraindicated.
**High doses (>750 mg/kg body weight)**	🚫 Should be avoided due to potential toxicity

✅: to be considered; 🚫: to pay attention.

## Abbreviations

The following abbreviations are used in this manuscript:

SCFA	Short-Chain Fatty Acids
IBD	Inflammatory Bowel Disease
IBS	Irritable Bowel Syndrome
ALD	Alcoholic Liver Disease
GIP	Glucose-dependent Insulinotropic Polypeptide
GLP-1	Glucagon-Like Peptide-1
PYY	Peptide YY
HDAC	Histone Deacetylase
IL-8	Interleukin-8
IL-12	Interleukin-12
IL-17	Interleukin-17
IL-22	Interleukin-22
CD8+	Cluster of Differentiation 8 Positive (Cytotoxic T cells)
γδ T cells	Gamma Delta T Cells
ILC3	Group 3 Innate Lymphoid Cells
MCT1	Monocarboxylate Transporter 1
NF-kB	Nuclear Factor kappa-light-chain-enhancer of activated B cells
ASD	Autism Spectrum Disorder
BBB	Blood–Brain Barrier
PA	Propionic Acidemia
PCCA/PCCB	Propionyl-CoA Carboxylase Subunit Alpha/Beta (genes involved in PA)
FFAR2	Free Fatty Acid Receptor 2 (also known as GPR43)
FFAR3	Free Fatty Acid Receptor 3 (also known as GPR41)
GPR43	G Protein-Coupled Receptor 43 (synonym of FFAR2)
GPR41	G Protein-Coupled Receptor 41 (synonym of FFAR3)
TLRs	Toll-Like Receptors
TD	Typical Development (control group in ASD studies)
